# Seasonality, long-term trends and co-occurrence of sharks in a top predator assemblage

**DOI:** 10.1371/journal.pone.0318011

**Published:** 2025-02-26

**Authors:** George P. Balchin, Anina Schuller, Isabella di Stefano, Michelle Robertson, Kym Pollard, William O. H. Hughes

**Affiliations:** 1 School of Life Sciences, University of Sussex, Brighton, United Kingdom; 2 Aqua Planet Dive Centre, Shelly Beach, KwaZulu Natal, South Africa; James Cook University, AUSTRALIA

## Abstract

Shark predator assemblages play an important role in the top-down processes that are vital to marine ecosystem functioning. Spatiotemporal partitioning of sharks due to seasonal movements or population changes may have significant consequences for the top-down effects, depending on the level of functional redundancy in the assemblage. However, long-term, co-occurrence data for sharks is hard to obtain and often lacking. Here we use citizen science data collected by professional scuba guides over seven years to model the seasonal and across-year temporal dynamics, and intraguild and trophic co-occurrence interactions, for an assemblage of six shark top predators (*Carcharhinus leucas*, *Carcharhinus obscurus*, *Carcharhinus limbatus*, *Carcharias taurus*, *Sphyrna lewini*, and *Galeocerdo cuvier*). The presence of all six study species were clearly seasonal and, in most cases, exhibited positive long-term trends across years. The seasonalities observed, combined with temporal co-occurrence analysis, suggests that dietary redundancy but temporal complementarity exists amongst the top predator assemblage. The study shows citizen science data collected by professional non-scientists is a cost-effective method for monitoring top predators and may be able to highlight potential predator-prey interactions worthy of further investigation.

## Introduction

Trophic downgrading is one of the most concerning anthropogenically driven forms of environmental degradation [1]. In marine systems, the continued impact of humans on the predatory guild threatens ecosystem functioning and services at a global scale [2,3]. Marine top predators such as sharks play key ecological roles that are vital to healthy ecosystem functioning and include both the direct effects of their predation and the indirect effects through modulation of prey behaviour [4]. Declines in predators or disruptions to their behaviour consequently reduce their suppression of lower trophic tiers and top-down regulation of food webs with cascading effects on ecosystem stability [4,5].

The occurrence of predators is not fixed in space or time and the spatiotemporal variation in predator occurrence is therefore fundamental to both understanding their ecology and managing their conservation. Many shark and other marine top predators exhibit seasonality in abundance and habitat occupancy [6–8]. These seasonal changes in habitat occupancy may be driven by changes in environmental conditions or food availability, or by migratory behaviour for reproduction [9,10]. They will inevitably result in a spatiotemporal pattern to the regulatory effects of the predator [11–13], for example top-down suppression of mesopredators can vary with the seasonal presence of top predators [14–16]. In addition, there is growing evidence that many shark top predators have exhibited substantial long-term temporal changes across years, principally population declines due to fishing activities or other anthropogenic factors [3]. These trends across years will be associated with spatiotemporal changes in the regulatory effects of the predator, such as the release of lower tier predators by top predator declines [17,18]. Seasonality will also impact the exposure of a species to threats and the effectiveness of conservation measures, while long-term patterns provide the key insight into declines or recoveries. Knowledge of both seasonal spatiotemporal patterns and long-term changes of shark populations is therefore essential for effective conservation management of predators, as well as for understanding their ecology. However, the data required to elucidate such patterns are often logistically challenging to obtain, particularly at local scales [19,20].

Another important contextual driver of top-down control is biodiversity, with higher levels of biodiversity generally being linked to ecosystem stability [21]. A single predator guild with multiple species may have functional redundancy, because the loss of one predator can be compensated by upregulation of others [22]. Understanding the dietary breadth and composition of the species within top predator assemblages is therefore important for understanding the resilience of systems to harmful trophic cascades [23]. Traditionally, stomach content analysis has been used to establish trophic relationships. However, this approach has many limitations: it is invasive, relies on satiation status, only provides a snapshot of their diet, has issues with prey species identification, and is confounded by prey species-specific variation in rate of digestion [24]. In addition to other modern methods such as stable isotope analysis and faecal DNA analysis, co-occurrence analysis of species observations has more recently been used to elucidate interspecific competition between predators and predator-prey interactions [25–29]. It has the drawback of only revealing correlations that may or may not represent ecological interactions, but as well as being non-invasive, has the advantage of not being dependent on physical interaction with rare, potentially vulnerable species with large home ranges. Co-occurrence analysis has also been used to investigate sightings data obtained through citizen science projects to not only assess the distribution of predators, but make inferences about the temporal nature of their interactions with prey as well [25,27,30].

Shark top predators are a primary attraction of many scuba diving tourism destinations and are often relatively easy to identify, making shark diving tourism a potentially rich source of citizen science data [30–33]. Although most shark diving tourism does not involve any manipulation, some operations use olfactory stimuli or provisioning of food to attract or maintain the interest of sharks. This can have behavioural or ecological implications in some, but not all, cases [34,35], and therefore needs to be accounted for in subsequent analysis. Shark diving tourism is a growing industry in South Africa that generates significant revenue [36], with it being particularly important in KwaZulu-Natal, on the eastern coast [37]. Many of the shark top predators in this area are of conservation concern [38], and KwaZulu-Natal therefore represents an opportunity to use the citizen science model to collect valuable data on the ecology, seasonality, and demography of these vulnerable species. Here we use a citizen science dataset of species recorded by professional guides working for a scuba diving tourism operator, across seven years at Protea Banks (KwaZulu-Natal, South Africa), a rocky reef that is a known hotspot for large sharks. We first use the data to elucidate the seasonality and long-term trends in the presence of six species of large pelagic and reef shark top predators, as well as to investigate the utility of the available extraneous data collected in a non-scientific context. In the second part of the study, we use co-occurrence analysis to map potential temporal associations, not only between the six study species, but also with all other species recorded, and identify potential predator-prey interactions.

## Methods

### Study site

KwaZulu-Natal runs down the eastern coast of South Africa and has complex reef systems occupying much of the coastline, which have high levels of shark biodiversity [39]. Protea Banks is a topologically complex, rocky reef habitat ca. 6 km long and 800 m wide, with an average depth of 30 m, which is situated approximately 8 km off Shelly Beach, KwaZulu-Natal (30° 49’ 58’‘ S, 30° 29’ 01’‘ E). It is notable as a scuba diving location due to its high likelihood of encounters with a diversity of sharks, principally oceanic blacktips (*Carcharhinus limbatus*), bull sharks (*Carcharhinus leucas*), ragged-tooth sharks (*Carcharias taurus*), scalloped hammerhead sharks (*Sphyrna lewini*), dusky sharks (*Carcharhinus obscurus*), and tiger sharks (*Galeocerdo cuvier*) [37]. Only two operators visited Protea Banks irregularly during most of the study period, so the level of disturbance or provisioning was consequently low and unlikely to alter shark behaviour on more than a transient and local scale.

### Data collection

Data were collected between 01/01/2013–31/12/2019 by professional scuba guides working for Aqua Planet Dive Centre. Data was collected during dives paid for by recreational scuba divers and therefore was sporadic with some gaps of several weeks, but across the study period dives occurred on 355 days of the calendar year, with average of 4.93 dives on those calendar days and at least 5 dives occurring on 181 calendar days across the seven years. The record of each dive included information on sea surface temperature (SST), dive type, areas of the dive site visited, estimated visibility and current strength, as well as a description of what species were seen. The species records were focussed on shark species, for which the number seen were recorded or estimated if > 5. The occurrence of other elasmobranch and teleost fish from multiple trophic tiers were also recorded for some of the dives (see S3 Table), including the number on some occasions or only presence on others. Visibility and current strength were recorded as ordinal factors (0–5 m, 6–10 m, 11–15 m, 16–20 m, 21–25 m, 26–30 m, 30+ m; and None, Mild, Medium, Normal, Medium-strong, Strong, respectively). Aqua Planet conducted two different dive types: midwater-provisioned and reef-nonprovisioned. Midwater-provisioned dives were drift dives, occurred exclusively in midwater at ca. 10–15 m depth, and used a bait drum filled with fresh, whole fish to chum the water and attract sharks to the area. Reef-nonprovisioned dives were also drift dives but occurred on the reef at ca. 20–30 m depth to allow observation of fauna on the reef from a relatively close distance. No provisioning or chumming occurred on reef-nonprovisioned dives, and provisioned dives occurred exclusively in midwater, therefore, dive type indicates the occurrence of provisioning as well as the approximate depth and habitat occupied during the dive. Other than visibility, survey effort data, such as dive duration or the number of divers present on each dive, were not collected.

### Data processing

The dataset included information from 2,132 dives. A total of 215 dives were excluded from the analysis: 199 dives completed in years with partial data (2012: 127 dives; 2020: 72 dives), 4 dives that were conducted at dives sites other than Protea Banks, and 12 dives that recorded extremely rare SST. Species sightings reported on the surface of the water were also excluded, as it could not be confirmed these occurred on a scuba dive. Temporal data such as day-of-year and day-of-study (a cumulative count of days in the study period) were added to allow seasonality, long-term trends and co-occurrence through time to be modelled, respectively. Absence data were then added, i.e., records of species not seen, therefore, each row of the dataset recorded the presence-absence of each species sighted on a scuba dive and all the other data associated with that dive. For the first part of the analysis, the dataset was then filtered for sightings of shark species of interest, resulting in 5,307 sightings across the 1,917 dives included in the analysis, and a dataset of 12,560 presence-absence records. This included 1,330 sightings of bull sharks, 216 sightings of dusky sharks, 1,914 sightings of oceanic blacktip sharks, 661 sightings of ragged-tooth sharks, 944 sightings of scalloped hammerhead sharks, and 242 sightings of tiger sharks. The second part of the analysis, focusing on co-occurrence, incorporated all species sightings into the analysis. As above, 215 dives were excluded, however the 401 midwater-provisioned dives were also excluded due to the confounding effect that baiting the water would have on any species co-occurrence observations. As a result, the co-occurrence analysis used a dataset with a total of 40,170 presence-absence records and 8,282 species sightings, across 25 different species and 1,516 scuba dives.

### Statistical analysis – Temporal trends and dive type

All statistical analyses were carried out in R (version 4.1.2) and R Studio (version 1.4.1) [40], using *α* = 0.05 as the critical value for significance. Due to abundance data being recorded predominately descriptively, rather than numerically, a binary response was used, reflecting the presence or absence of each species. As such, the predicted response was the probability of observing a species on a scuba dive, herein called ‘presence’. Due to a highly non-linear relationship with both day-of-year and day-of-study, as well as the long-term nature of the dataset and relatively even spread of the data through time, a generalised additive modelling approach was taken (GAM: R package *mgcv*) [41]. A separate model for each species was built using a binomial error structure and logit link function, as well as with Reduced Maximum Likelihood (REML) to avoid overfitting. Non-linearity of the day-of-study response was further investigated through visualisation of residuals from a GAM in which the day-of-study predictor had been removed, to ensure the non-linearity of the relationship persisted when other variables of interest had been controlled for. SST was excluded as a model predictor due to its clear relationship with day-of-year, as was evident in the raw data plots (S2 Fig). A thin-plate regression spline was used to smooth the effect of day-of-study, and a cyclic cubic regression spline was used to smooth the effect of day-of-year and accommodate its cyclic nature. The effect of dive type was included as a factor. Visibility was treated as a random effect to control for survey bias; current strength was included as a random effect for those species where the raw data indicated a relationship (S4 and S5 Figs) to control for survey effort (changes in dive type due to ocean conditions that effect presence, for example, strong currents) or any associated shift in shark behaviour. Diagnostic plots were visually inspected to ensure that the assumptions of the modelling process were met (S3 Fig). A stepwise model simplification process was followed, using ANOVA to compare models and remove nonsignificant terms. Whenever significant patterns were detected in model residuals for a particular predictor, the number of base functions (*k*) were increased, within the constraints of the degrees of freedom available, to ensure these patterns could not be accounted for by increasing model complexity. If there was no effect on residual patterns, then *k* was returned to its default value. All covariates were checked for concurvity with a threshold of 0.8.

### Statistical analysis – Co-occurrence

Presence-absence data for all 25 recorded predators and prey were used to determine temporal co-occurrence both between the study species themselves (bull sharks, dusky sharks, oceanic blacktip sharks, ragged-tooth sharks, scalloped hammerhead sharks, and tiger sharks) and between the study species and other species observed during the study, including potential prey species. A co-occurrence was defined as the presence of 2 species on a single scuba dive. Initially, the data were correlated for all combinations of the study species and potential prey species by producing a Spearman’s rank correlation matrix, using a false discovery rate procedure to correct for multiple comparisons. Potential associations were then further explored using the *cooccur* package in R, to build a probabilistic model of species co-occurrence, by comparing the observed co-occurrence to that which would be expected randomly [42]. The *cooccur* package is designed to estimate non-random co-occurrence at independent sites, it compares the co-occurrence of two species against that which would be expected at random and infers associations on this basis. However, here *cooccur* has been manipulated to estimate co-occurrence through time by substituting sites for each individual day of the study period during which at least one scuba dive occurred (1,086 days). Circular network plots were produced to visualise the strength, significance and direction of potential associations between the species.

## Results

At least one of the shark species was seen on 1,818 of the 1,917 dives analysed (94.8%). The most commonly sighted species was the oceanic blacktip shark, seen on 1,520 dives (79.3%). Bull sharks were seen on 1,100 dives (57.4%), scalloped hammerhead sharks on 690 dives (36.0%), ragged-tooth sharks on 523 dives (27.3%), tiger sharks on 225 dives (11.7%), and dusky sharks on 200 dives (10.4%). Of the 1,917 dives, 1,552 (81.0%) were reef-nonprovisioned and 365 (19.0%) were midwater-provisioned.

### Seasonality

**Fig 1:**
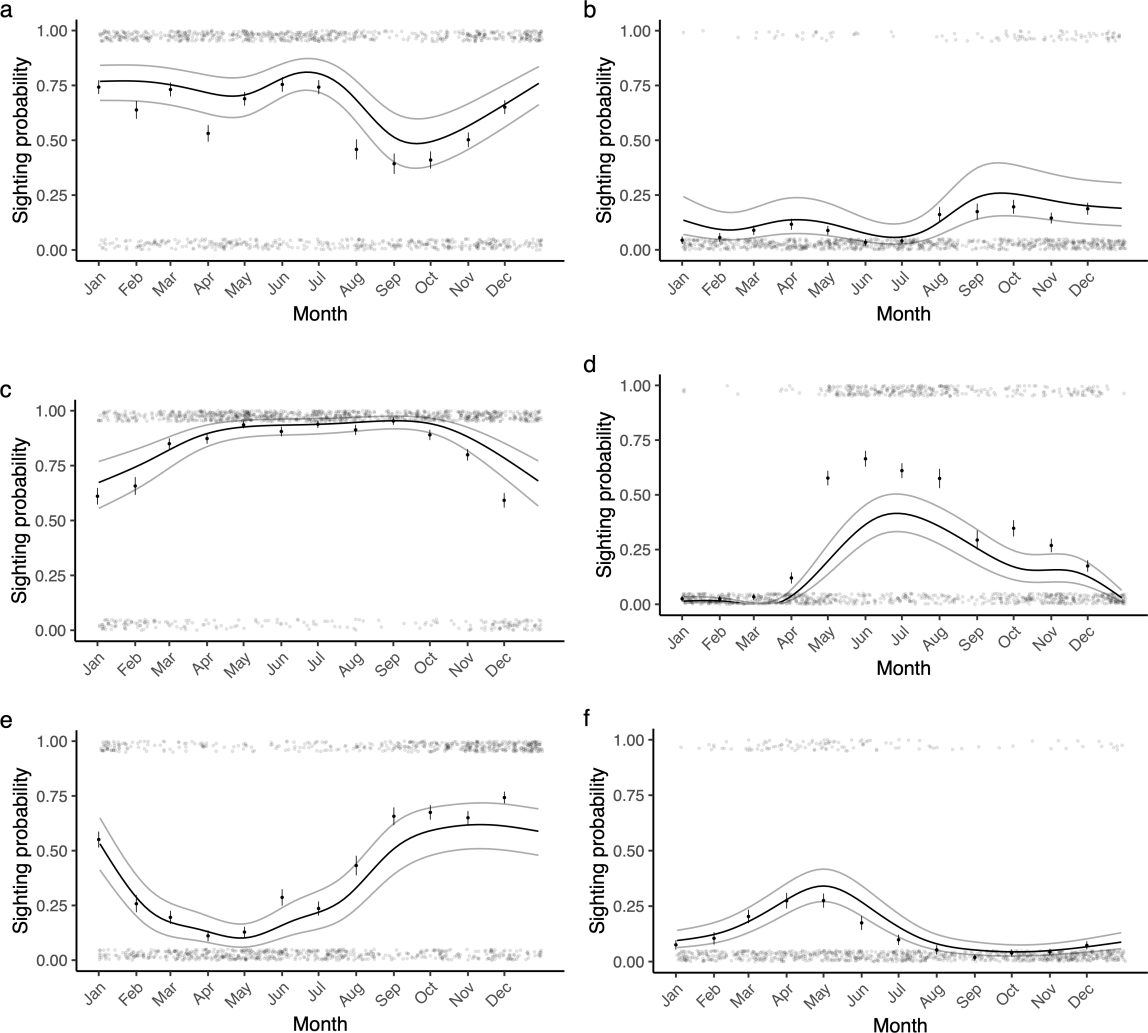
The seasonality of shark sightings. The probability of sighting a shark on a scuba dive for a) bull sharks, b) dusky sharks, c) oceanic blacktip sharks, d) ragged-tooth sharks, e) scalloped hammerhead sharks, and f) tiger sharks, at Protea Banks, South Africa. The translucent points show the raw data (plus jitter), the solid points and bars show the mean ± SE for each month, the black line shows the model estimate, and the grey lines show the 95% CI.

The sightings of all study species exhibited significant seasonality, with a clear peak in the season, no obvious signs of substantial multimodality, and interspecific differences in the timing, extent, and duration of the seasonal peaks (Fig 1). Bull sharks and oceanic blacktip sharks were common throughout most of the year, with both seen on the majority of dives, excluding three months (August-October) for bull sharks (respectively: edf = 6.45, χ^2^ = 128.6, p < 0.001, Fig 1a; edf = 5.32, χ^2^ = 222.1, p < 0.001, Fig 1c). Ragged-tooth sharks, scalloped hammerhead sharks, and tiger sharks showed the strongest seasonality, with periods of relative absence followed by increases in presence to 30–50% during the peak of the season (respectively: edf = 6.71, χ^2^ = 1,044.9, p < 0.001, Fig 1d; edf = 6.45, χ^2^ = 877.4, p < 0.001, Fig 1e; edf = 4.32, χ^2^ = 114.0, p < 0.001, Fig 1f). The least common species, the dusky shark, had the most muted seasonality, and was observed on less than 10% of dives for most the year, peaking towards the end of the year at 19.6% (edf = 5.79, χ^2^ = 58.2, p < 0.001, Fig 1b).

### Trends across years

**Fig 2:**
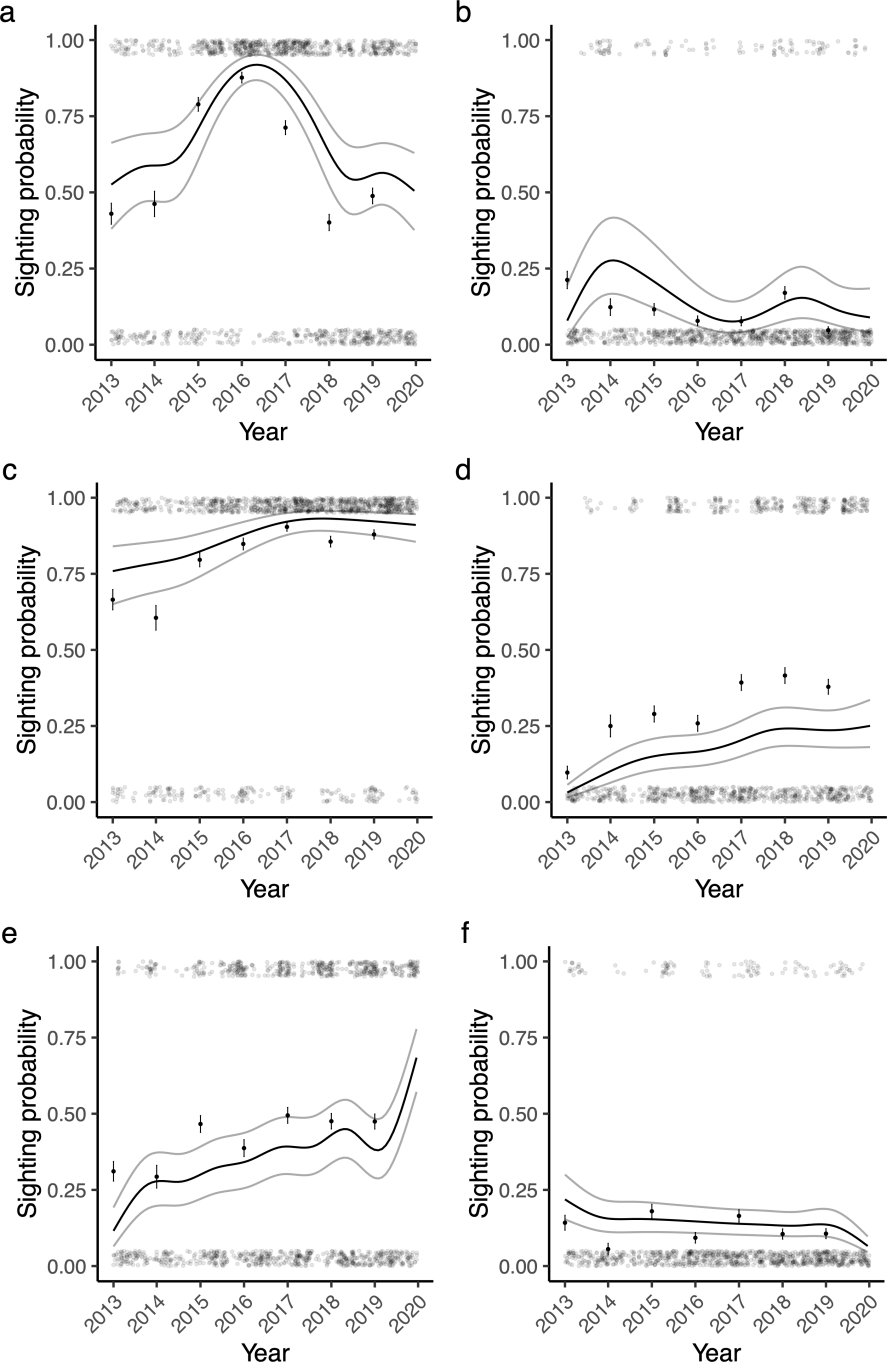
Long-term temporal trends in shark sightings. The probability of sighting a shark on a scuba dive through time for a) bull sharks, b) dusky sharks, c) oceanic blacktip sharks, d) ragged-tooth sharks, e) scalloped hammerhead sharks, and f) tiger sharks, at Protea Banks, South Africa. The translucent points show the raw data (plus jitter), the solid points and bars show the mean ± SE across years, the black line shows the model estimate, and the grey lines show the 95% CI.

Five of six shark species exhibited significant changes in presence across years. The most extreme change was observed in bull sharks, sightings of which increased from 43–46% of dives in 2013–2014 to over 70–88% of dives in 2015–2017, before declining to 40–49% in 2018–2019 (edf = 7.53, χ^2^ = 283.1, p < 0.001, Fig 2a). Sightings of oceanic blacktip sharks, ragged-tooth sharks, and scalloped hammerhead sharks increased by 21.4%, 28.2%, and 16.3%, respectively, over the course of the study (respectively: edf = 5.00, χ^2^ = 95.4, p < 0.001, Fig 2c; edf = 4.52, χ^2^ = 87.3, p < 0.001, Fig 2d; edf = 2.06, χ^2^ = 47.9, p < 0.001, Fig 2e). Increases in ragged-tooth sharks and scalloped hammerhead sharks, were consistent throughout the study period, while sightings of oceanic blacktip sharks levelled off towards the end of the study period (Fig 2). The presence of dusky sharks declined reasonably consistently from 21.3% in 2013 to 4.7% in 2019 (edf = 4.45, χ^2^ = 50.7, p < 0.001, Fig 2b). There was no significant change in tiger shark sightings across years (edf = 1.00, χ^2^ = 2.45, p = 0.12, Fig 2f).

### Dive type

**Fig 3:**
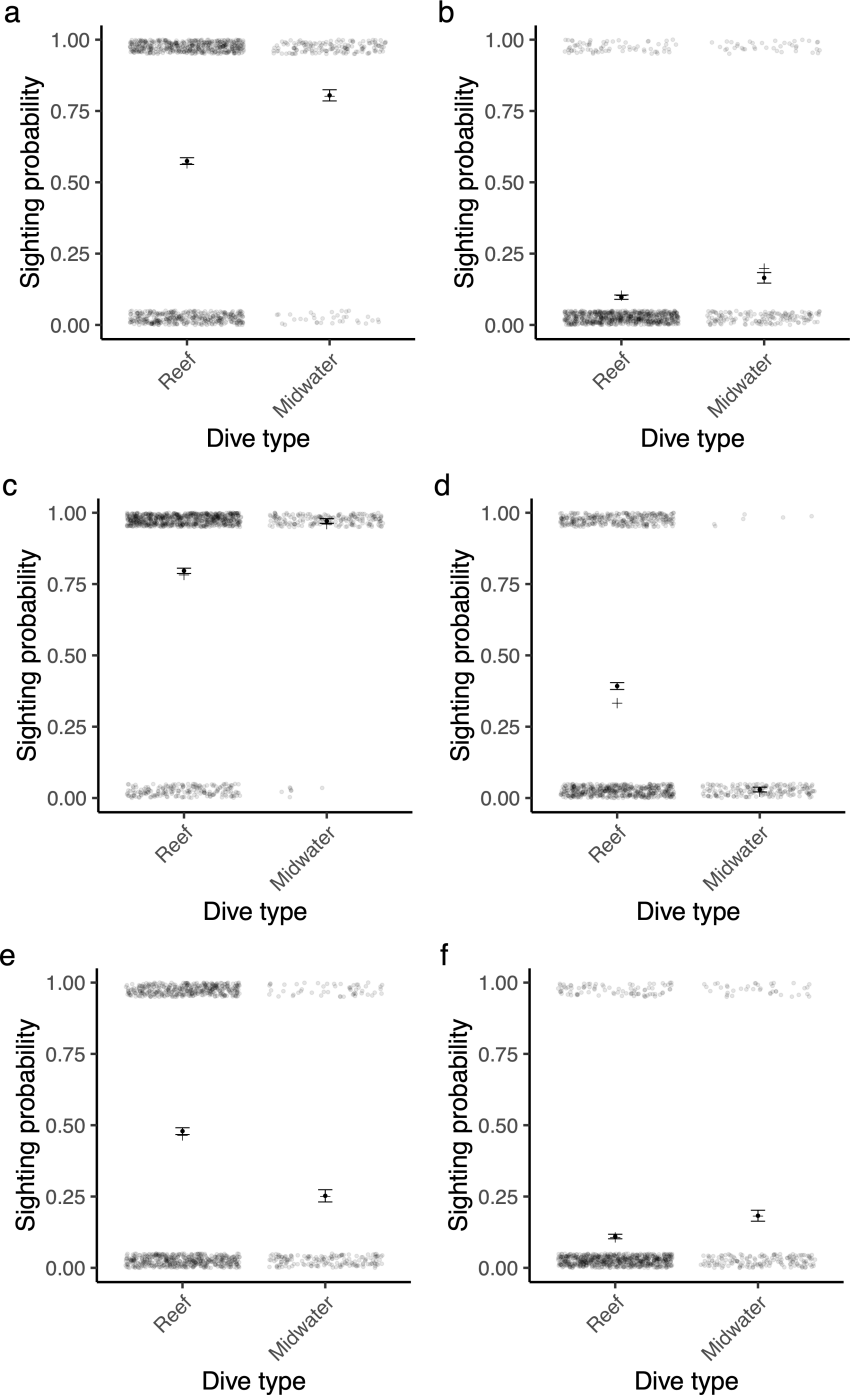
The effect of dive type on shark sightings. The probability of sighting a shark on either a midwater-provisioned or reef-nonprovisioned scuba dive for a) bull sharks, b) dusky sharks, c) oceanic blacktip sharks, d) ragged-tooth sharks, e) scalloped hammerhead sharks, and f) tiger sharks against dive type (either provisioned and in midwater, or nonprovisioned and close to the reef), at Protea Banks, South Africa. The translucent points show the raw data (plus jitter), the solid points and bars show the mean ± SE, and the crosses show the model estimate.

The presence of all study species was significantly affected by dive type, but the direction of this effect was species-specific. Bull sharks, dusky sharks, oceanic blacktip sharks, and tiger sharks were more frequently observed on midwater-provisioned dives than on reef-nonprovisioned dives (respectively: Estimate = −1.40, *z* = −9.27, p < 0.001, Fig 3a; Estimate = −0.84, *z* = −4.90, p < 0.001, Fig 3b; Estimate = −2.20, *z* = −6.92, Fig 3c, p < 0.001; Estimate = 0.67, *z* = 4.17, p < 0.001, Fig 3f). In contrast, ragged-tooth sharks and scalloped hammerhead sharks were seen more frequently on reef-nonprovisioned dives than on midwater-provisioned dives (respectively: Estimate = 4.13, *z* = 12.5, p < 0.001, Fig 3d; Estimate = 1.32, *z* = 8.99, p < 0.001, Fig 3e).

### Random factors and model fit

Visibility and current strength (where included) explained a significant amount of the variance in the data for all study species, except tiger sharks (S1 Table, S4 and S5 Figs). The variance explained by each random effect differed between the study species. Visibility explained 38.6% (± 31.6–52.7% CI) of the variance in bull shark presence, 38.9% (± 30.1–61.9% CI) of the variance in dusky shark presence, 37.4% (± 30.3–53.8% CI) of the variance in oceanic blacktip shark presence, 33.6% (± 27.7–52.9% CI) of the variance in ragged-tooth shark presence, 40.2% (± 32.5–55.4% CI) of the variance in scalloped hammerhead shark presence, and 25.1% (± 25–100% CI) of the variance in tiger shark presence. Current strength explained 47% (± 36–67% CI) of the variance in ragged-tooth shark presence, and 42.7% (± 33.8–59.8% CI) of the variance in scalloped hammerhead shark presence. With the possible exception of ragged-tooth sharks, visual assessment of model predictions indicated that they were in line with the observed data (Figs 1 and 2). Formal assessment of model fit varied for each study species; the best fitting model, for ragged-tooth sharks, had an *R*^2^ of 0.49, whereas the worst fitting model, for tiger sharks, had an *R*^2^ of 0.09.

### Co-occurrence

**Fig 4:**
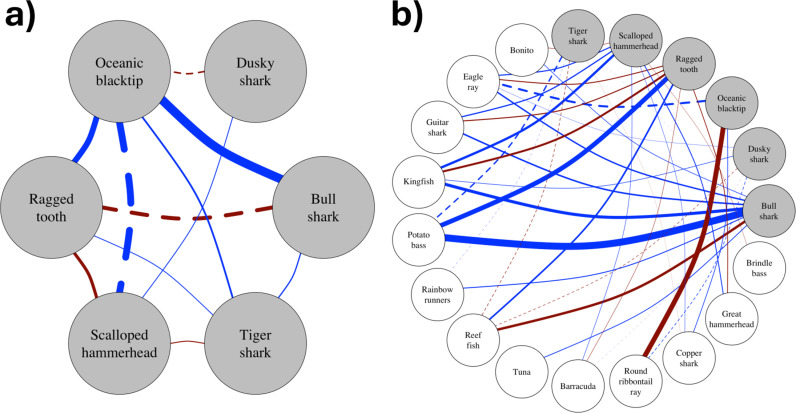
Network of species associations between a) focal shark species and b) focal shark species and other species. Each node represents a species, with the lines between them representing potential associations. The thickness of the line is scaled according to the likelihood of those species co-occurring (‘co-prob; S1 and S2 Tables). Lines show whether associations were positive (blue) or negative (red), and significant (solid) or nonsignificant (dashed) based on Spearman’s correlation matrix corrected for multiple comparisons using False Discovery Rate.

For each shark species, significant associations were identified both with other top-predator shark species (Fig 4a), and with potential prey species (Fig 4b). In all cases, a significant association identified by multiple comparisons, was further validated by a probabilistic model of species co-occurrence (S2 and S3 Tables). Of the shark species, significant positive associations were identified between bull sharks and oceanic blacktip sharks, bull sharks and tiger sharks, dusky sharks and oceanic blacktip sharks, dusky sharks and scalloped hammerhead sharks, oceanic blacktip sharks and ragged-tooth sharks, oceanic blacktip sharks and tiger sharks, and ragged-tooth sharks and tiger sharks. Negative associations were identified between bull sharks and ragged-tooth sharks, and scalloped hammerhead sharks and tiger sharks. There was high overlap between the associations of the study species with potential prey species, with all six shark top predator species showing significant associations with other smaller elasmobranchs, and all but oceanic blacktip sharks with various teleost species (Fig 4b). The diversity and number of species associations differed among the study species, from between three associations in the case of dusky sharks to 13 associations in the case of scalloped hammerhead sharks.

## Discussion

The results demonstrated that all six species of shark studied showed strong seasonalities in occurrence at Protea Banks, and all but one species also showed significant changes across years, with interspecific differences in the timing, direction and magnitude of the effects. The co-occurrence results showed significant species associations that in some cases matched known dietary relationships. Basic data routinely collected by divers, such as visibility, current strength, and dive type, proved useful to control for variation in survey effort and other factors that affect presence. In combination, these findings support the value of shark diving tourism as a source of citizen science-derived monitoring data.

All six of the focal shark species exhibited significant seasonality in presence and in each case, the seasonality pattern was species-specific. The occurrence of tiger sharks was highest in March-June, ragged-tooth sharks in May-August, and scalloped hammerheads in September-January, whilst the occurrence of the more common bull sharks and oceanic blacktip sharks was highest in December-July and March-November, respectively. These seasonal patterns could be explained by either smaller scale movements associated with habitat selection [6,43,44], or large scale migrations of the kind observed in many shark species including some of those studied here, driven by environmental conditions, food availability or migration for reproduction [45–49]. The strongest seasonal signals were shown by ragged-tooth sharks and scalloped hammerheads. Mature female ragged-tooth sharks undergo a biennial migration along the South African coast associated with mating in the area of the study site in, it is believed, October-November, gestation in the warmer waters to the north and parturition in the cooler waters of the Eastern Cape in September to November; juveniles remain relatively close to their nursery grounds and the movement patterns of mature males are unclear [50]. The high occurrence of ragged-tooth sharks we found at Protea Banks in May-November overlaps with the mating and southward migration periods of mature females, and also included mature males (WOHH pers. obs.), suggesting that this may be a key area in the life-history of the species. The finding that scalloped hammerheads were most common during the summer (September-January) was in keeping with previous findings for this species in the region that together support a migratory movement centred on the core Transkei region and possibly related to sea temperature [45,51,52]. Importantly, the interspecific differences in seasonal patterns seen here imply reduced intraguild competition and greater complementarity in resource partitioning, resulting in potentially year-round top-down control [53]. The seasonality of shark presence also has important implications for conservation management, such as fisheries restrictions. Interspecific complementarity resulting in year-round presence of sharks precludes identification of a temporal period in which none of the species would be affected, but seasonal fisheries restrictions could be seasonally targeted on particular species of greatest conservation concern.

All of the study species, except tiger sharks, showed a significant change in sightings across years. Any change at the start or end of the study period should be interpreted with caution, especially in instances where seasonal increases or decreases overlap with the start or end of the calendar year, as was the case with scalloped hammerhead sharks. However, several species still showed general long-term changes that were not explained by confounding seasonal effects. The increases in sightings of oceanic blacktips and ragged-tooth sharks could be due to increases in occurrence, but could also be due to the dive guides becoming better at attracting (in the case of oceanic blacktips on midwater-provisioned dives) or finding (in the case of ragged-tooth sharks in the reef caves) sharks over the course of the seven years. Scalloped hammerhead sharks rarely interact with bait on midwater-provisioned dives and are not found in predictable locations on the reef, so the increase in the presence of this species most likely reflects a genuine increase in abundance. The increases in these three species, and the stability of tiger shark sightings, are nevertheless reassuring from a conservation perspective and may reflect the protection which has been afforded to these species at local and global levels [38,54]. In contrast, sightings of bull sharks and dusky sharks peaked in 2016 and 2014 respectively, before declining over the rest of the study period. This could reflect competitive exclusion on provisioned dives by the more active oceanic blacktip sharks, local flux in resources at Protea Banks, or a wider change in abundance.

The results provided some useful insights into the co-occurrence of species, illustrating the complexity of potential species associations. There were high levels of overlap in associations between the shark study species and various species of teleosts and smaller elasmobranchs that other studies have shown to be potential prey [45,55–57]. This could potentially suggest some level of dietary overlap and redundancy amongst the shark top predator assemblage at Protea Banks. The dietary overlaps included some that have been previously identified, for example between dusky sharks, scalloped hammerhead sharks and oceanic blacktips [56]. However, co-occurrence, or lack thereof, does not necessarily indicate an ecological interaction [58], and the usefulness of the analysis is therefore primarily in identifying potential interactions for further investigation. In addition, the results were limited in only recording sightings as occurring at some point during the 45–60 min scuba dive. For future citizen-science studies, increasing the temporal resolution of the data or specifically recording simultaneous co-occurrences that are observed would add significant value to the data.

The study shows some of the ways in which the scientific value of citizen science data can be readily strengthened. Despite the large size of the dataset, there were inevitably data gaps across the study period, but the modelling approach used was successful at overcoming these to produce reliable predictions of seasonal and long-term population dynamics. Despite data being collected by experienced dive guides, there will inevitably have been survey bias, but the incorporation of visibility and current strength into the models proved valuable to control for this. For example, sightings of bull sharks and scalloped hammerhead sharks correlated positively with visibility, likely because they are relatively timid and so more often sighted at a distance. Factoring in dive type was particularly important, with ragged-tooth sharks being exclusively observed on reef-nonprovisioned dives, while oceanic blacktips, bull sharks, tiger sharks and dusky sharks were predominantly observed on midwater-provisioned dives, in keeping with their respective foraging ecologies [59,60]. Although the nature of the data collection meant that the effects of provisioning and habitat could not be separated, the two dive types provided data on a greater range of species than would have been possible using either dive type alone. Many recreational shark dives can vary in the nature and extent to which the dive is focussed on shark interactions, and incorporation of dive type at even a simple level may therefore be similarly valuable in other citizen science data. The provisioning of sharks, as well as other effects of the tourism operation such as diver or boat presence, has the potential to alter shark behaviour, and so needs to be considered when interpreting the results and assessing the cost-benefit trade-off of a citizen science study.

The results illustrate the value of scuba diving tourism for the investigation and long-term monitoring of shark populations [30,32]. Such data will inevitably have limitations due to the pragmatic compromise with the tourism operation, but these can be mitigated through use of diving professionals for data collection, and modelling that incorporates environmental variables. Shark diving citizen science can provide valuable long-term data for conservation management, whilst also benefiting the diving operators, thereby increasing the potential conservation benefits of shark diving tourism [31–33]. Furthermore, shark diving tourism increases the economic worth of shark populations, adding to its value as a conservation tool [36]. Given the importance of shark top predator assemblages for ecosystem functioning and the anthropogenic threats they face, all avenues of monitoring and research data should be utilised to ensure multi-scale temporal and spatial trends are documented to aid their conservation and recovery.

## Supporting information

S1 Table The effects of visibility and current strength on shark sightings. Model coefficients from generalised additive model predicting the effect of visibility and current strength (current strength not included in all models) on the presence (the probability of sighting a shark on a SCUBA dive) for bull sharks, dusky sharks, oceanic blacktip sharks, ragged-tooth sharks, scalloped hammerhead sharks, and tiger sharks, between 2013–2020, at Protea Banks, South Africa.
(PDF)

S2 Table Associations between focal shark species. For each pair of species, the incidence that each species (Sp 1, Sp 2) was observed on scuba dives between 2013 and 2019 at Protea Banks, South Africa, the correlation coefficient (r) and p-value corrected for the false discovery rate of the association (p (fdr)). The table also shows the number (co_obs), probability (co_prob), and expected number (co_exp) of co-occurrences, and the p-values for negative or positive associations indicated by a probabilistic model of non-random occurrence (p_neg, p_pos).
(PDF)

S3 Table Associations between focal shark speices and other species. For each pair of species, the incidence that each species (Sp 1, Sp 2) was observed on scuba dives between 2013 and 2019 at Protea Banks, South Africa, the correlation coefficient (r) and p-value corrected for the false discovery rate of the association (p (fdr)). The table also shows the number (co_obs), probability (co_prob), and expected number (co_exp) of co-occurrences, and the p-values for negative or positive associations indicated by a probabilistic model of non-random occurrence (p_neg, p_pos).
(PDF)

S1 Fig The long-term trends in shark presence after all other model terms of interest have been controlled for (residual plot). Data are of the probability of sighting a shark on a SCUBA dive for bull sharks, dusky sharks, blacktip sharks, ragged-tooth sharks, scalloped hammerhead sharks, and tiger sharks at Protea Banks, South Africa. The solid black points and bars show the mean ± SE estimated from the raw data.
(PDF)

S2 Fig Seasonal fluctuation in sea temperature. Data are for sea surface temperature at Protea Banks, South Africa, between 2013 and 2020. The solid black points and bars show the mean ± SE estimated from the raw data.
(PDF)

S3 Fig Model diagnostic plots. Diagnostics are for when predicting presence (the probability of sighting a shark on a SCUBA dive) for a) bull sharks, b) dusky sharks, c) blacktip sharks, d) ragged-tooth sharks, e) scalloped hammerhead sharks, and f) tiger sharks at Protea Banks, South Africa.
(PDF)

S4 Fig The effect of visibility on shark sightings. Presence (the probability of sighting a shark on a SCUBA dive) of a) of bull sharks, b) dusky sharks, c) oceanic blacktip sharks, d) ragged-tooth sharks, e) scalloped hammerhead sharks, and d) tiger sharks against water visibility (m). Data collected between 2013–2020, at Protea Banks, South Africa. The solid points and bars show the mean ± SE; the translucent jittered points show the raw data; the crosses show the model prediction.
(PDF)

S5 Fig The effect of current strength on shark sightings. Presence (the probability of sighting a shark on a SCUBA dive) of a) of bull sharks, b) dusky sharks, c) oceanic blacktip sharks, d) ragged-tooth sharks, e) scalloped hammerhead sharks, and d) tiger sharks against current strength. Data collected between 2013–2020, at Protea Banks, South Africa. The solid points and bars show the mean ± SE; the translucent jittered points show the raw data; the crosses show the model prediction.
(PDF)
