# Supplementary material for: Seasonality, long-term trends and co-occurrence of sharks in a top predator assemblage
Source: PLoS One. 2025 Feb 26;20(2):e0318011. doi: 10.1371/journal.pone.0318011 (PMC11864520; doi:10.1371/journal.pone.0318011)
Supplement: S1 Table — Model coefficients from generalised additive model predicting the effect of visibility and current strength (current strength not included in all models) on the presence (the probability of sighting a shark on a SCUBA dive) for bull sharks, dusky sharks, oceanic blacktip sharks, ragged-tooth sharks, scalloped hammerhead sharks, and tiger sharks, between 2013–2020, at Protea Banks, South Africa. (PDF) [file pone.0318011.s001.pdf]

S1 Table

Bull shark				
**Smooth terms**	**edf**	**Ref.*df***	χ^2	***p***
s(visibility)	5.01	6	39.59	<0.0001
**Dusky shark**				
**Smooth terms**	**edf**	**Ref.*df***	χ^2	***p***
s(visibility)	4.577	6	15.45	0.00289
**Oceanic blacktip shark**				
**Smooth terms**	**edf**	**Ref.*df***	χ^2	***p***
s(visibility)	4.688	6	21.06	<0.0001
**Ragged tooth shark**				
**Smooth terms**	**edf**	**Ref.*df***	χ^2	***p***
s(visibility)	3.579	6	13.09	0.00512
s(current)	4.717	5	97.31	<0.0001
**Scalloped hammerhead**				
**Smooth terms**	**edf**	**Ref.*df***	χ^2	***p***
s(visibility)	5.191	6	53.91	<0.0001
s(current)	4.762	5	107.88	<0.0001
**Tiger shark**				
**Smooth terms**	**edf**	**Ref.*df***	χ^2	***p***
s(visibility)	0.001133	6	0.001	0.471
