# Supplementary material for: Seasonality, long-term trends and co-occurrence of sharks in a top predator assemblage
Source: PLoS One. 2025 Feb 26;20(2):e0318011. doi: 10.1371/journal.pone.0318011 (PMC11864520; doi:10.1371/journal.pone.0318011)
Supplement: S2 Table — For each pair of species, the incidence that each species (Sp 1, Sp 2) was observed on scuba dives between 2013 and 2019 at Protea Banks, South Africa, the correlation coefficient (r) and p-value corrected for the false discovery rate of the association (p (fdr)). The table also shows the number (co_obs), probability (co_prob), and expected number (co_exp) of co-occurrences, and the p-values for negative or positive associations indicated by a probabilistic model of non-random occurrence (p_neg, p_pos). (PDF) [file pone.0318011.s002.pdf]

S1 Table

Species 1	Species 2	Sp 1	Sp 2	r	p (fdr)	co_obs	co_prob	co_exp	p_neg	p_pos
bull shark	dusky shark									
bull shark	oceanic blacktip	775	1130	0.092	0.002	608	0.381	577.7	1.000	<0.001
bull shark	ragged tooth	775	511	-0.054	0.101	242	0.172	261.2	0.021	0.984
bull shark	scalloped hammerhead									
bull shark	tiger shark	775	154	0.097	0.001	101	0.052	78.7	1.000	<0.001
dusky shark	oceanic blacktip	134	1130	0.049	0.145	109	0.066	99.9	0.980	0.034
dusky shark	ragged tooth									
dusky shark	scalloped hammerhead	134	603	0.117	<0.001	78	0.035	53.3	1.000	<0.001
dusky shark	tiger shark									
oceanic blacktip	ragged tooth	1130	511	0.116	<0.001	417	0.251	380.9	1.000	<0.001
oceanic blacktip	scalloped hammerhead	1130	603	0.060	0.059	469	0.296	449.5	0.992	0.0106
oceanic blacktip	tiger shark	1130	154	0.091	0.002	133	0.076	114.8	1.000	<0.001
ragged tooth	scalloped hammerhead	511	603	-0.135	<0.001	156	0.134	203.3	0.000	1
ragged tooth	tiger shark	511	154	0.097	0.001	73	0.034	51.9	1.000	<0.001
scalloped hammerhead	tiger shark	603	154	-0.081	0.006	43	0.04	61.3	0.001	0.9996
