# Supplementary material for: Seasonality, long-term trends and co-occurrence of sharks in a top predator assemblage
Source: PLoS One. 2025 Feb 26;20(2):e0318011. doi: 10.1371/journal.pone.0318011 (PMC11864520; doi:10.1371/journal.pone.0318011)
Supplement: S3 Table — For each pair of species, the incidence that each species (Sp 1, Sp 2) was observed on scuba dives between 2013 and 2019 at Protea Banks, South Africa, the correlation coefficient (r) and p-value corrected for the false discovery rate of the association (p (fdr)). The table also shows the number (co_obs), probability (co_prob), and expected number (co_exp) of co-occurrences, and the p-values for negative or positive associations indicated by a probabilistic model of non-random occurrence (p_neg, p_pos). (PDF) [file pone.0318011.s003.pdf]

S2 Table

Species 1	Species 2	Sp 1	Sp 2	r	p (fdr)	co_obs	co_prob	co_exp	p_neg	p_pos
bull shark	bonito	775	69	0.068	0.03	46	0.023	35.3	0.997	0.006
bull shark	eagle ray	775	169	0.065	0.038	102	0.057	86.4	0.996	0.007
bull shark	guitar shark	775	185	0.086	0.004	116	0.062	94.6	1.000	<0.001
bull shark	kingfish	775	364	0.086	0.004	214	0.123	186.1	1.000	<0.001
bull shark	potato bass	775	839	0.077	0.011	458	0.283	428.9	0.999	0.002
bull shark	rainbow runners	775	107	0.125	<0.001	79	0.036	54.7	1.000	<0.001
bull shark	reef fish	775	298	-0.194	<0.001	94	0.1	152.3	0.000	1.000
bull shark	tuna	775	93	0.063	0.047	59	0.031	47.5	0.995	0.009
dusky shark	barracuda	134	66	0.059	0.069	11	0.004	5.8	0.989	0.026
dusky shark	eagle ray	134	169	0.089	0.003	27	0.01	14.9	1.000	0.001
dusky shark	kingfish	134	364	0.075	0.014	46	0.021	32.2	0.998	0.003
dusky shark	reef fish	134	298	-0.055	0.095	17	0.017	26.3	0.019	0.990
dusky shark	round ribbontail ray	134	398	0.047	0.169	44	0.023	35.2	0.970	0.046
oceanic blacktip	copper shark	1130	53	0.087	0.004	50	0.026	39.5	1.000	<0.001
oceanic blacktip	eagle ray	1130	169	0.053	0.105	137	0.083	126	0.987	0.022
oceanic blacktip	round ribbontail ray	1130	398	-0.075	0.014	275	0.196	296.7	0.003	0.998
ragged tooth	barracuda	511	66	-0.070	0.024	12	0.015	22.2	0.003	0.999
ragged tooth	eagle ray	511	169	-0.128	<0.001	28	0.038	57	<0.001	1.000
ragged tooth	great hammerhead	511	130	-0.094	0.002	25	0.029	43.8	<0.001	1.000
ragged tooth	guitar shark	511	185	-0.210	<0.001	13	0.041	62.4	<0.001	1.000
ragged tooth	kingfish	511	364	-0.159	<0.001	74	0.081	122.7	<0.001	1.000
ragged tooth	potato bass	511	839	0.152	<0.001	337	0.187	282.8	1.000	<0.001
ragged tooth	reef fish	511	298	0.100	0.001	129	0.066	100.4	1.000	<0.001
scalloped hammerhead	barracuda	603	66	0.117	<0.001	44	0.017	26.3	1.000	<0.001
scalloped hammerhead	bonito	603	69	-0.093	0.002	13	0.018	27.4	<0.001	1.000
scalloped hammerhead	brindle bass	603	40	-0.083	0.005	6	0.01	15.9	0.001	1.000
scalloped hammerhead	copper shark	603	53	0.190	<0.001	47	0.014	21.1	1.000	<0.001
scalloped hammerhead	eagle ray	603	169	0.162	<0.001	105	0.044	67.2	1.000	<0.001
scalloped hammerhead	great hammerhead	603	130	0.127	<0.001	78	0.034	51.7	1.000	<0.001
scalloped hammerhead	guitar shark	603	185	0.187	<0.001	119	0.049	73.6	1.000	<0.001
scalloped hammerhead	kingfish	603	364	0.086	0.004	172	0.096	144.8	1.000	0.001
tiger shark	brindle bass	154	40	0.054	0.101	8	0.003	4.1	0.984	0.043
tiger shark	copper shark	154	53	-0.064	0.042	0	0.004	5.4	0.003	1.000
tiger shark	potato bass	154	839	0.056	0.083	98	0.056	85.2	0.989	0.017
tiger shark	rainbow runners	154	107	0.052	0.108	17	0.007	10.9	0.982	0.036
tiger shark	reef fish	154	298	-0.062	0.051	19	0.02	30.3	0.008	0.996
tiger shark	reef fish	154	298	-0.062	0.051	19	0.02	30.3	0.008	0.996
