# Supplementary figures and images for: Seasonality, long-term trends and co-occurrence of sharks in a top predator assemblage
Source: PLoS One. 2025 Feb 26;20(2):e0318011. doi: 10.1371/journal.pone.0318011 (PMC11864520; doi:10.1371/journal.pone.0318011)

**S1 Fig.**

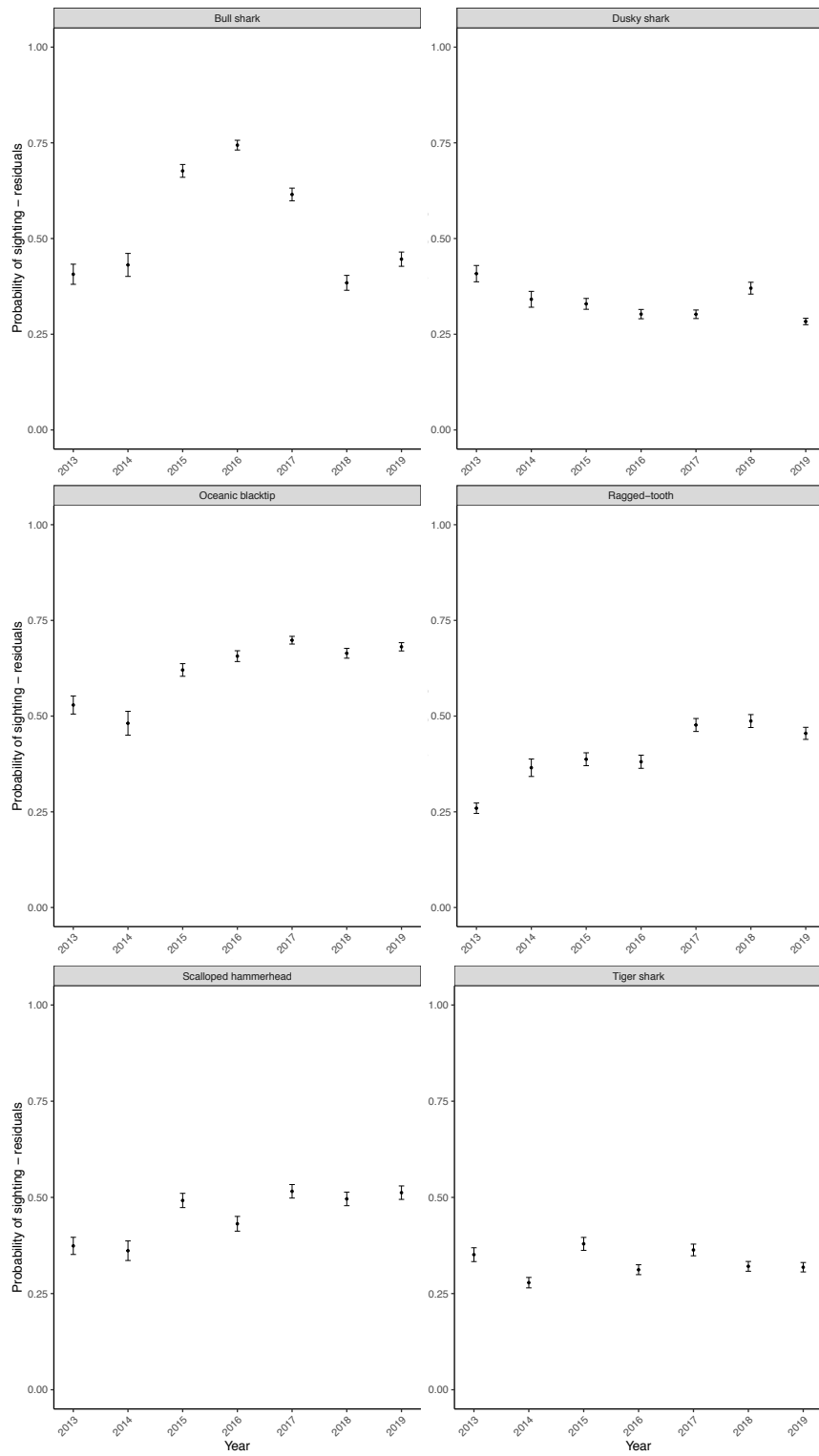

Supplement: S1 Fig — Data are of the probability of sighting a shark on a SCUBA dive for bull sharks, dusky sharks, blacktip sharks, ragged-tooth sharks, scalloped hammerhead sharks, and tiger sharks at Protea Banks, South Africa. The solid black points and bars show the mean ± SE estimated from the raw data. (PDF) [file pone.0318011.s004.pdf]

**S2 Fig.**

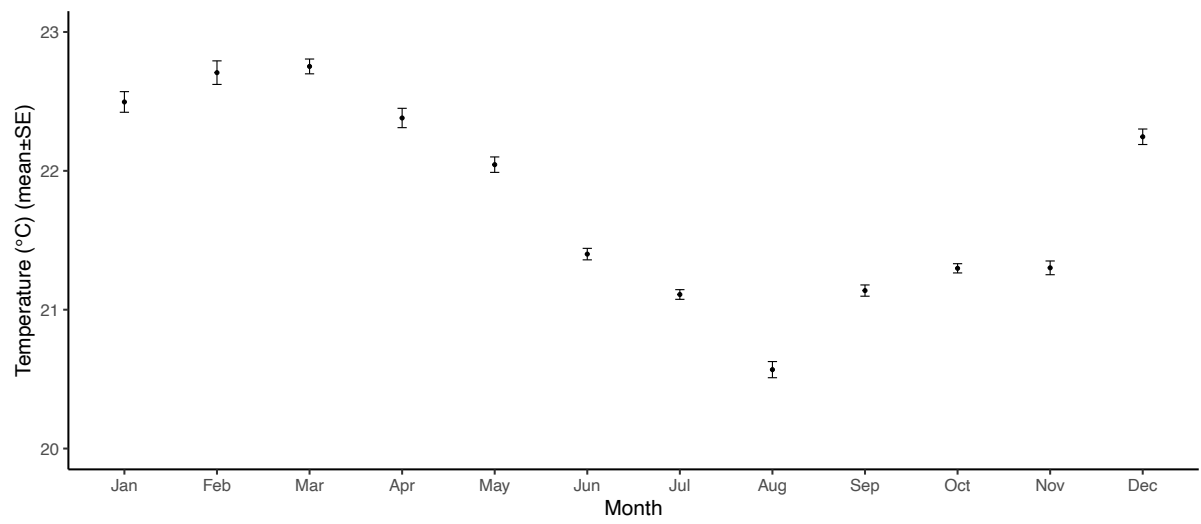

Supplement: S2 Fig — Data are for sea surface temperature at Protea Banks, South Africa, between 2013 and 2020. The solid black points and bars show the mean ± SE estimated from the raw data. (PDF) [file pone.0318011.s005.pdf]

**S3 Fig.**

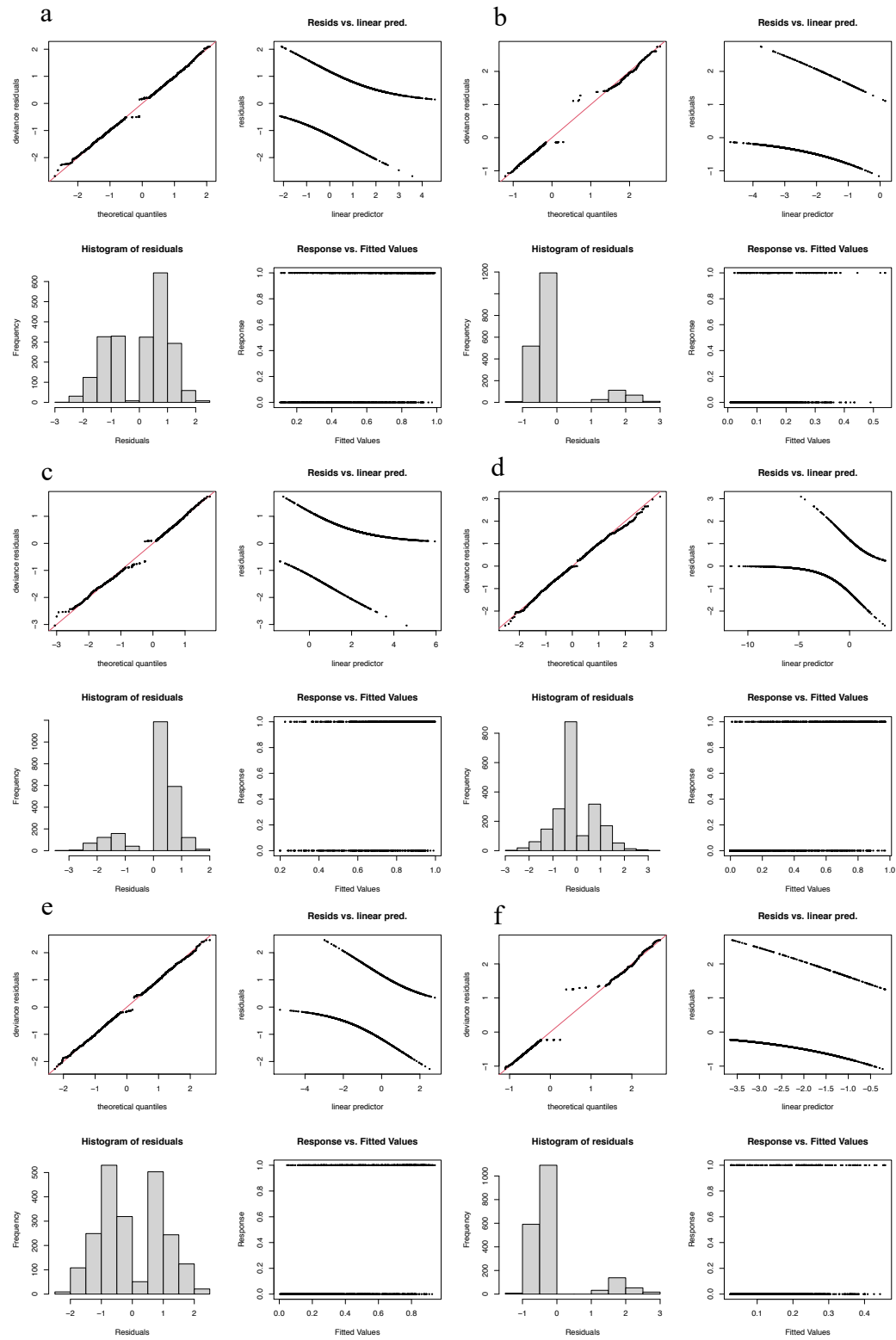

Supplement: S3 Fig — Diagnostics are for when predicting presence (the probability of sighting a shark on a SCUBA dive) for a) bull sharks, b) dusky sharks, c) blacktip sharks, d) ragged-tooth sharks, e) scalloped hammerhead sharks, and f) tiger sharks at Protea Banks, South Africa. (PDF) [file pone.0318011.s006.pdf]

**S4 Figure**

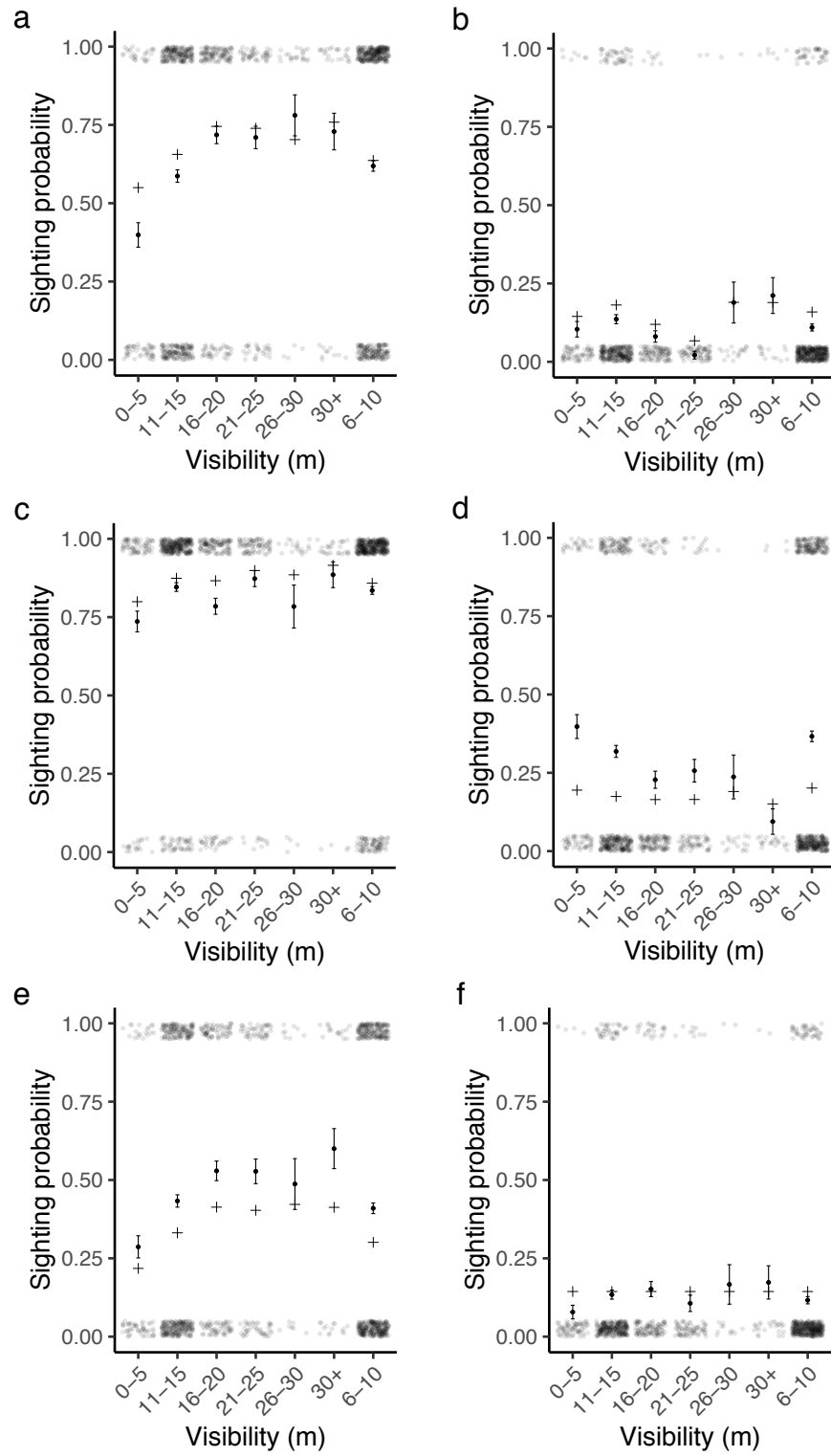

Supplement: S4 Fig — Presence (the probability of sighting a shark on a SCUBA dive) of a) of bull sharks, b) dusky sharks, c) oceanic blacktip sharks, d) ragged-tooth sharks, e) scalloped hammerhead sharks, and d) tiger sharks against water visibility (m). Data collected between 2013–2020, at Protea Banks, South Africa. The solid points and bars show the mean ± SE; the translucent jittered points show the raw data; the crosses show the model prediction. (PDF) [file pone.0318011.s007.pdf]

S5 Figure

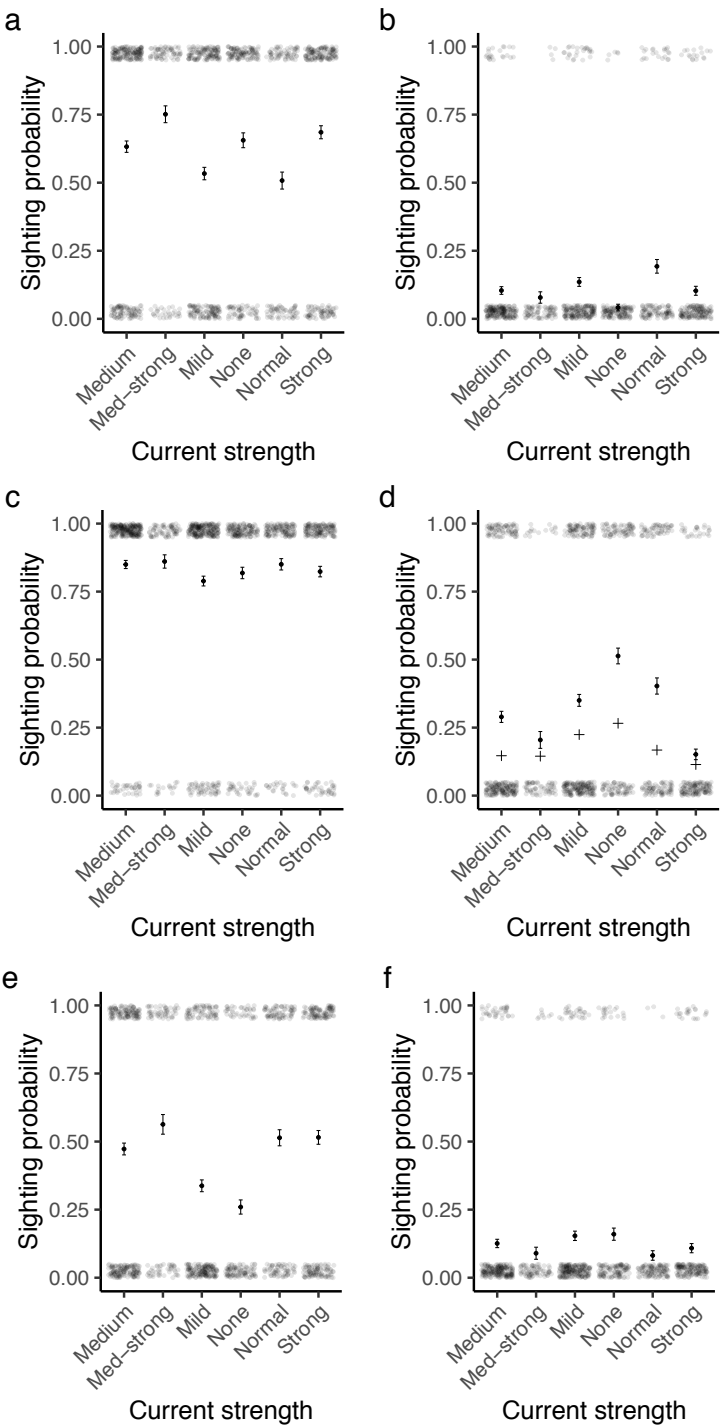

Supplement: S5 Fig — Presence (the probability of sighting a shark on a SCUBA dive) of a) of bull sharks, b) dusky sharks, c) oceanic blacktip sharks, d) ragged-tooth sharks, e) scalloped hammerhead sharks, and d) tiger sharks against current strength. Data collected between 2013–2020, at Protea Banks, South Africa. The solid points and bars show the mean ± SE; the translucent jittered points show the raw data; the crosses show the model prediction. (PDF) [file pone.0318011.s008.pdf]
